# DNA Protection by an Aronia Juice-Based Food Supplement

**DOI:** 10.3390/antiox10060857

**Published:** 2021-05-27

**Authors:** Tamara Bakuradze, Peter Meiser, Jens Galan, Elke Richling

**Affiliations:** 1 Division of Food Chemistry & Toxicology, Department of Chemistry, Technische Universität Kaiserslautern, Erwin-Schrödinger-Str. 52, 67663 Kaiserslautern, Germany; bakuradze@chemie.uni-kl.de; 2 URSAPHARM Arzneimittel GmbH, Industriestr. 35, 66129 Saarbrücken, Germany; peter.meiser@ursapharm.de; 3 Hochgewanne 23, 67269 Grünstadt, Germany; mail@Galan.info

**Keywords:** aronia, DNA strand breaks, comet assay, H_2_O_2_, isolated peripheral lymphocytes

## Abstract

Background: This study investigated the effects of an aronia juice-based food supplement on background and total DNA strand breaks in whole blood, and on H_2_O_2_-induced DNA strand breaks in isolated peripheral blood lymphocytes. Methods: Ninety-one healthy volunteers were randomly selected to consume either the food supplement (2 × 25 mL drinking ampules, *n* = 45) or no supplement (*n* = 46) daily for eight weeks. Results: Background DNA strand breaks decreased significantly after four and eight weeks of supplement consumption, compared to baseline (*p* < 0.05), but the overall effect was low, and neither group showed a decrease in total DNA strand breaks. Conversely, supplement consumption clearly reduced H_2_O_2_-induced DNA strand breaks ex vivo (*p* < 0.001), with statistically significant reductions after four and eight weeks, compared to the control group (*p* < 0.05). Conclusions: Thus, although consuming antioxidant supplements might produce only marginal immediate benefits under healthy conditions, potential preventive effects warrant further investigation.

## 1. Introduction

Increased formation of reactive oxygen species (ROS) is associated with degenerative diseases like atherosclerosis, Alzheimer’s disease, Parkinson’s disease, and cancer [1]. The damaging effects of ROS on cellular biomolecules, including lipids, proteins, and DNA, are leading causes of the development and progression of these diseases.

Anthocyanins and other phenolic compounds are major phytochemicals in fruits and vegetables that exhibit powerful antioxidant and DNA-protective activities both in vitro and in vivo [2]. Of the flavonoid-rich fruits, chokeberry (*Aronia melanocarpa*) is distinguished for its significantly high anthocyanin (641–1959 mg/100 g dry weight) and ascorbic acid content (13–270 mg/Kg fresh weight) [3,4]. Anthocyanins can scavenge diverse ROS or induce expression of enzymes like superoxide dismutase (SOD), catalase (CAT), and glutathione peroxidase (GPx) which depend on antioxidant/electrophile response elements [5,6]. Several intervention studies reported that consumption of anthocyanin-rich products may protect human DNA integrity and is associated with health benefits [7,8,9,10], where DNA integrity may be assessed by the level of background or total DNA damage.

Breaks in background DNA strands result from endogenous exposure to DNA-damaging agents and/or incomplete DNA repair. In addition to background strand breaks, total DNA damage includes damage induced by ROS and other electrophilic compounds (e.g., aldehydes, acrylamide, aromatic hydrocarbon quinones) which lead to lesions that can be detected with specific repair enzymes like formamidopyrimidine glycosylase (FPG) [11]. A recent intervention study reported a significant reduction in background and total DNA strand breaks in whole blood of healthy volunteers after eight weeks intake of an anthocyanin-rich fruit juice (750 mL/day, corresponding to 274.5 mg/L total anthocyanins) [7]. Similarly, four weeks consumption of a red fruit juice (700 mL/day, corresponding to 197.9 mg/L total anthocyanins) led to a decrease in oxidative DNA damage in healthy humans [8], and consumption of a blueberry drink (25 g of wild blueberry freeze-dried powder in 250 mL water, containing ca 375 mg total anthocyanins) over six weeks protected lymphocytes from hydrogen peroxide (H_2_O_2_)-induced and total DNA damage, respectively [12]. Controlled studies on the consumption of polyphenol-rich coffee (750 mL/day, containing 10.2 mg/g caffeoylquinic acids) also yielded similar results [13,14]. However, translation of these findings into a daily routine is unsuitable because the daily volumes of test drinks required were unreasonably large and regular daily intake of high amounts of sugar or caffeine may have negative health impacts [15,16].

Therefore, we aimed to close the resulting gap between degenerative disease etiology and potential anthocyanin treatment by developing a concentrated polyphenol-rich food supplement that combines chokeberry (*Aronia melanocarpa*, hereafter referred to as ‘aronia’) juice concentrate with micronutrients like zinc and selenium (Table 1), elements known to boost the endogenous antioxidative defense [4,17,18,19]. Recent testing of this food supplement in a small (*n* = 10) uncontrolled pilot intervention study over a four-week period indicated the potential for DNA-protective effects, even at low doses (2 × 25 mL/day). These results did not achieve statistical significance and are of limited validity since the study had no control group [20].

The goal of the present prospective, randomized, controlled, human intervention study was thus to scrutinize the long-term effects of the newly-developed anthocyanin- and micronutrient-rich food supplement on DNA integrity in whole blood cells under controlled conditions. Total DNA strand breaks in isolated peripheral lymphocytes were also analyzed after H_2_O_2_ treatment to monitor alterations in cell sensitivity to ROS-induced DNA-damage with and without supplement intervention.

## 2. Materials and Methods

### 2.1. Chemicals

All chemicals used in the presented research were of analytical grade. Histopaque-1077 was obtained from Sigma–Aldrich (Steinheim, Germany) and RPMI 1640 medium from Life Technologies GmbH/ThermoFisher Scientific (Darmstadt, Germany); H_2_O_2_ from VWR International GmbH (Darmstadt, Germany) and GelRed from Biotium (Fremont, CA, USA).

### 2.2. Food Supplement Supply

The aronia juice-based food supplement (brand name: aronia+ IMMUN) was supplied by Ursapharm Arzneimittel GmbH, Saarbruecken, Germany. One drinking ampule (25 mL) contains aronia juice (aronia juice produced from aronia concentrate), sucrose, citric acid, sodium benzoate, potassium sorbate, zinc gluconate, natural aroma, niacin, pantothenic acid, vitamin B1, vitamin B2, vitamin B6, sodium selenite, and vitamin D (Table 1). The aronia juice concentrate used in the food supplement is gained from pressed aronia fruits (*Aronia melanocarpa*, Poland). The aronia concentrate was previously analyzed in detail [21], revealing a high polyphenolic content (total polyphenols determined by Folin Ciocalteu method: 3.3%, corresponding to 222 mg/ampule); flavonoids, calculated as hyperoside: 0.07% (corresponding to 4.7 mg/ampule); anthocyanins, calculated as cyanidine-3-*O*-glucoside: 0.6%(corresponding to 40.5 mg/ampule); proanthocyanidins, calculated according to Prior et al. [22] 1% (corresponding to 67.5 mg/ampule), using 4-dimethylaminocinnamaldehyde (DMAC) colorimetric method with commercially available standard. Regarding the colorless polyphenols, the most abundant compounds in aronia juice concentrate were found to be hydroxycinnamic acid derivatives [21].

### 2.3. Subjects and Study Design

This study was designed as a prospective, randomized, controlled, parallel group clinical trial. The study was approved by the ethics committee of Rhineland-Palatinate, Mainz, Germany [No. 2018-13854], and was performed in accordance with the ethical standards stipulated by the 1964 Declaration of Helsinki and its later amendments. This study was registered in the German Clinical Trial Register (DRKS00016656).

The goal was to enroll 100 healthy male and female volunteers who fulfilled the following inclusion/exclusion criteria: age 20–50 years, BMI 19–32 kg/m^2^, non-smokers, no alcohol consumption on a regular basis, non-vegetarians, did not practice excessive sports, did not take pharmaceutical drugs or food supplements for one week prior to study participation and throughout the study, had no pre-existing disease, was not pregnant, was not simultaneously participating in another study, and did not donate blood during the observation period. We recruited male and female volunteers of the age 20 and 50 years to achieve a homogenous healthy collective. All volunteers had to provide written informed consent before enrollment into the study. After enrollment, all volunteers underwent standard medical health checks, including a questionnaire, blood pressure and anthropometric measurements, and standard clinical blood biochemistry tests. Subsequently, the volunteers were randomly assigned to receive either the food supplement (test group) or no intervention (control group).

**Figure 1:**
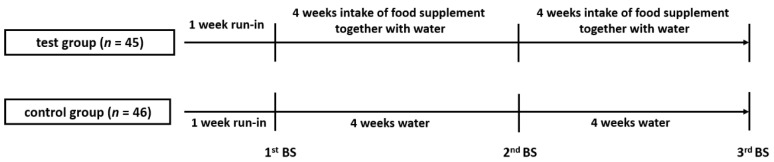
Study Design and Conduct: after a one-week run-in phase, healthy participants assigned to the test group consumed one ampule of the food supplement together with 200 mL of water twice daily for eight weeks. Participants assigned to the control group consumed water only. Blood sampling (BS) was performed after the run-in phase (1^st^ BS), four weeks into the intervention phase (2^nd^ BS), and after completion of the intervention phase (i.e., eight weeks, 3^rd^ BS).

After a one-week run-in period involving standardized intake of 200 mL of water each morning and evening, test group volunteers consumed two ampules of the food supplement (Table 1) together with 200 mL of water, one between 8 a.m. and 10 a.m. and the other between 4 and 6 p.m. each day, over a period of eight weeks (Figure 1). The control group continued consuming the standardized volume of water (200 mL) each morning and evening throughout the entire study period. Time (day and hour) of daily food supplement consumption was recorded for compliance control. Volunteers were instructed to maintain their normal dietary and lifestyle habits during the study but to abstain from consuming large amounts of polyphenol-rich foods. Furthermore, volunteers were also instructed to record their daily health status and subjective wellbeing, including incidences of illnesses or disease, during the study. General state of health was assessed daily on a scale from 0 (best grade) to 10 (worst grade).

Venous blood samples (10 mL) were collected after the one-week run-in period (before starting the intervention) and after four weeks and eight weeks of the intervention. Blood sampling after four weeks and eight weeks was performed 1 h after intake of the food supplement (test group) or water (control group), respectively. Modulation in background and total DNA-strand breaks was determined from the whole blood samples collected. Additionally, the experiment involving H_2_O_2_-induced DNA damage (“H_2_O_2_ challenge”) was conducted using peripheral blood lymphocytes isolated from the subgroups of male volunteers only due to the laborious experimental setup.

### 2.4. Blood Sample Collection and Isolation of Human Peripheral Blood Lymphocytes (PBL)

Venous blood was collected in EDTA-coated tubes and the samples were immediately used for the comet assay for whole blood. Human peripheral blood lymphocytes (PBLs) were isolated by centrifugation with Histopaque-1077. Briefly, 7 mL of freshly-collected human blood was layered onto 7 mL of Histopaque-1077 and continuously centrifuged at room temperature at 400× *g* for 25 min. PBLs were extracted from the cloudy middle layer, transferred to 10 mL RPMI 1640 medium supplemented with 10% fetal calf serum (FCS) and 1% penicillin/streptomycin, and tempered at 37 °C. Next, this cell suspension was centrifuged for 10 min at 250× *g*. The resulting supernatant was discarded, and the pellet was re-dissolved in 6 mL RPMI 1640 medium and centrifuged again. After centrifugation, the resulting supernatant was discarded, and the pellet re-dissolved in 1 mL RPMI 1640 medium. Immediately afterwards, the PBLs were analyzed using the comet assay.

### 2.5. Body Weight Measurement

Body weight and height (digital precision scale, Seca delta 707, Hamburg, Germany) of the volunteers were measured, and their BMI (kg/m^2^) was calculated.

### 2.6. Single Cell Gel Electrophoresis Experiments (Comet Assay)

#### 2.6.1. Comet Assay with Whole Blood Samples

Alkaline single-cell gel electrophoresis was performed according to Collins et al. [23], with slight modifications as previously reported [20]. Briefly, aliquots of blood (6 µL) were mixed with low-melting agarose (65 µL), applied onto a precoated microscope slide, and subjected to lysis (1 h, at 4 °C). Thereafter, the slides were washed three times in enzyme buffer, drained, and covered with 50 μL of either enzyme buffer or formamidopyrimidine-DNA glycosylase (FPG enzyme) to differentiate between background and total DNA strand breaks (i.e., background strand breaks and oxidized bases). After DNA unwinding (pH 13.5, 20 min, 4 °C) and horizontal gel electrophoresis (20 min, 25 V, 300 mA), the slides were washed, stained with GelRed, and analyzed using a fluorescence microscope (Imager, A1, filter set 15, Zeiss, Germany) and computerized image analysis (Comet Assay II, Perceptive Instruments, Suffolk, GB), scoring 2 × 50 cells per slide. DNA migration was directly expressed as the mean tail intensity (TI%) of two gels.

#### 2.6.2. Comet Assay in Isolated PBLs after H_2_O_2_ Challenge

Immediately after isolation of lymphocytes, 2 × 50,000 isolated PBLs were centrifuged, and the pellet was mixed with low-melting agarose and applied onto a precoated microscope slide. Next, the slides were exposed to H_2_O_2_ (50 µM) on ice for 5 min, according to previous publications [24,25], to allow monitoring of alterations in cell sensitivity towards ROS. After the treatment, the cells were subjected to lysis, washed (three times with enzyme buffer), then incubated in 50 µL FPG solution for 30 min at 37 °C. After DNA unwinding and horizontal gel electrophoresis, slides were washed, stained with GelRed, and analyzed using a fluorescence microscope (see above).

The cell viability was determined after isolation of lymphocytes as well as after the incubation of lymphocytes with 50 µM H_2_O_2_.

The mean viability of lymphocytes after isolation was 99.2 ± 0.6% and after incubation with H_2_O_2_ 98.9 ± 0.7%.

#### 2.6.3. Statistical Analysis

Sample size estimation was based on the results of a pilot study performed in April 2017 that investigated the effects of four weeks of daily aronia juice consumption. Assuming a standard deviation like that of the pilot study (i.e., 0.16) for the primary endpoint of this investigation, a sample size estimate of 46 samples per group was anticipated to achieve a significance for a difference of 0.11 when comparing the test and control groups. The power was set to 0.9 and the level of significance to 0.5 using a two-sided test. Accounting for a dropout rate of about 10%, a sample size of 50 samples per group was chosen for the study.

Results of the experiments conducted are reported as mean and SD. All hypotheses were tested against the two-sided alternative. The normality hypothesis was tested using the Shapiro–Wilk test. Analysis of Covariance (ANCOVA) was used to compare the control and intervention groups by time point. If the normality assumption was rejected, the ANCOVA was based on Blom transformed ranks. Changes from baseline within groups were analyzed using the paired *t*-test for normal distributions and the Wilcoxon-signed-rank test for non-normal distributions. *p*-values below 0.05 were regarded as significant

## 3. Results

### 3.1. Demographic Data, Baseline Characteristics, and Health Status of Volunteer Participants

Demographic data from the healthy volunteers included in the study are provided in Table 2. Informed consent was obtained from all healthy volunteers prior to study participation. Overall, 99 volunteers participated in the study, with 50 randomized to the test group for food supplement consumption and 49 assigned to the control group.

**Figure 2:**
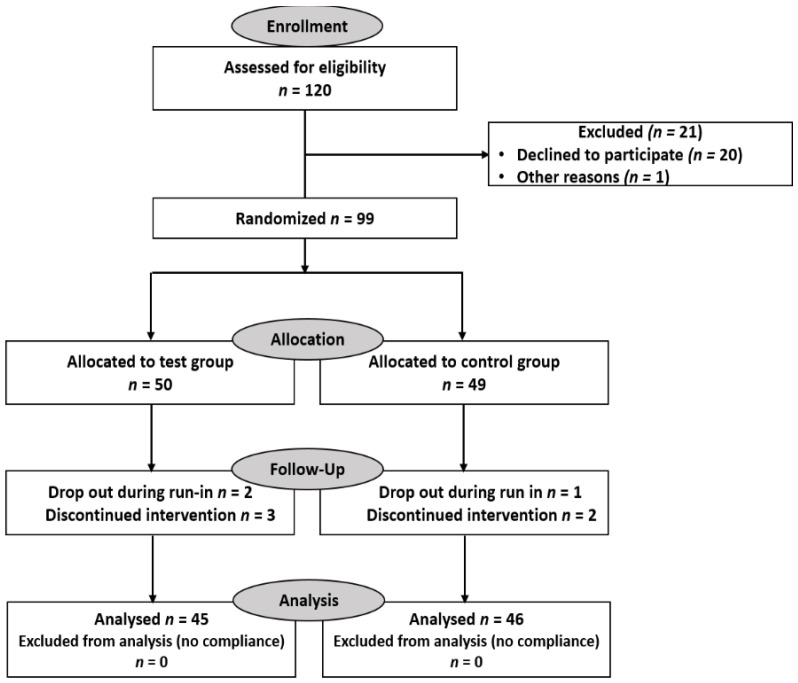
CONSORT flow diagram.

Altogether, 91 volunteers finished the eight-week intervention period (Figure 1 and Figure 2).

Each day during the study, participants assessed and recorded their general health status and subjective wellbeing. General state of health was assessed using a scale ranging from 0 (best grade) to 10 (worst grade), and assessments from the test and control groups yielded average values of 3.28 ± 1.88 and 3.69 ± 1.70, respectively, over the eight-week observation period (between-group differences were not statistically significant). Three volunteers from the control group reported illnesses occurring during the observation period (common cold; common cold and gastrointestinal infection; and erythema nodosum).

### 3.2. Aronia Juice-Based Food Supplement Lowers Whole Blood Background DNA Strand Breaks in Healthy Adults

**Figure 3:**
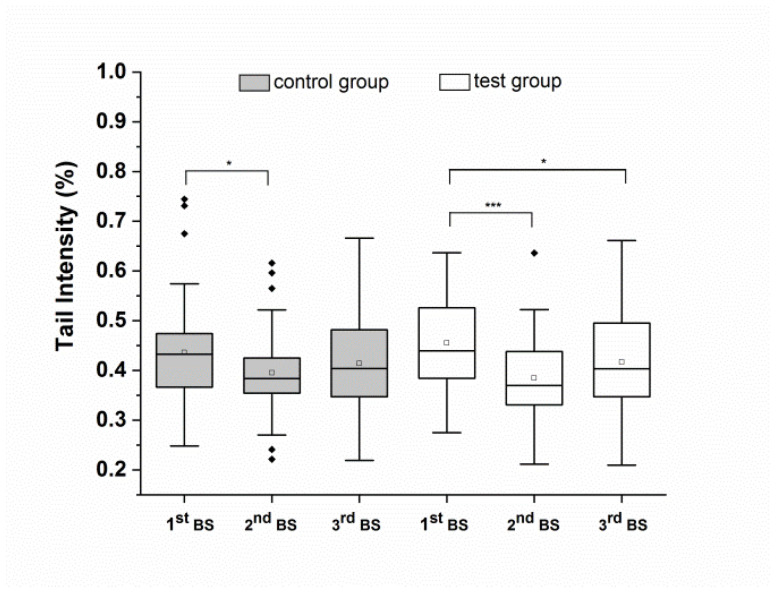
Background DNA strand breaks (without FPG treatment) in whole blood after the run-in phase (1^st^ BS), and after four weeks (2^nd^ BS) and eight weeks (3^rd^ BS) of the intervention. Data are expressed as mean tail intensity [%] and SD. Squares represent mean values; horizontal lines within the boxes represent median values; rhombuses represent outliers. BS: blood sampling. * *p* < 0.05, *** *p* < 0.001.

The effects of food supplement consumption for eight weeks on background and total DNA strand breaks in the whole blood of healthy volunteers, when compared to the effects of no supplementation, were prespecified to establish the primary endpoints of the study. Results of background DNA strand breaks are presented in Figure 3 and are summarized also as numerical values in Table 3.

Compared to baseline, food supplement consumption (test group) significantly reduced background DNA strand breaks after four (*p* < 0.001) and eight (*p* < 0.05) weeks. Background DNA strand breaks decreased with no supplement (control group) after four weeks but remained unchanged at the end of the study period. No significant differences in background DNA strand breaks were observed between the test and control groups.

**Figure 4:**
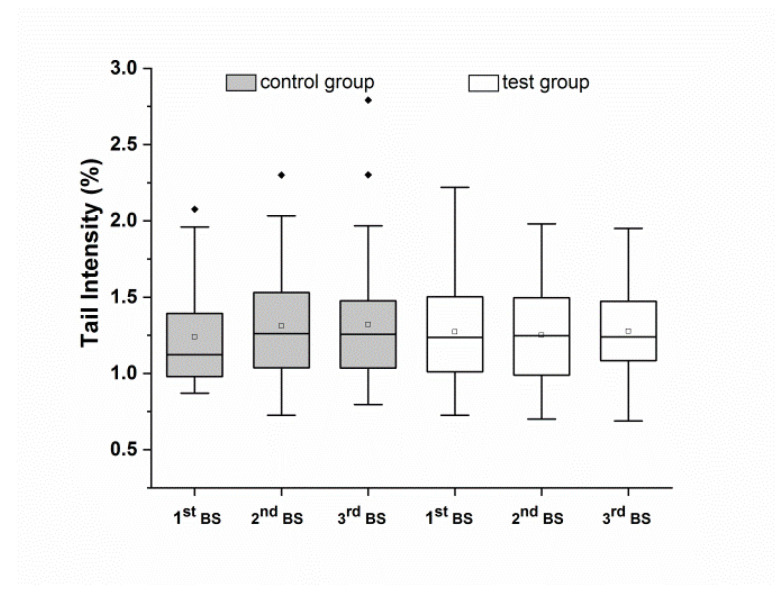
Total DNA strand breaks (with FPG treatment) in whole blood cells after run-in (1^st^ BS), and after four weeks (2^nd^ BS) and eight weeks (3^rd^ BS) of the intervention. Data are expressed as mean tail intensity [%] and SD. Squares represent mean values; horizontal lines within the boxes represent median values; rhombuses represent outliers. BS: blood sampling.

Furthermore, total DNA strand breaks (i.e., background + FPG-sensitive sites) showed no significant changes over the observation period in either group (Figure 4), and no significant differences between groups at the end of the observation period. No gender-specific effects were observed for the investigations of background and total DNA strand breaks.

Table 4 represents the summarized results as numeric values.

### 3.3. Intake of the Food Supplement Prevents Total DNA Damaging of Isolated Peripheral Blood Lymphocytes after H_2_O_2_ Challenge

**Figure 5:**
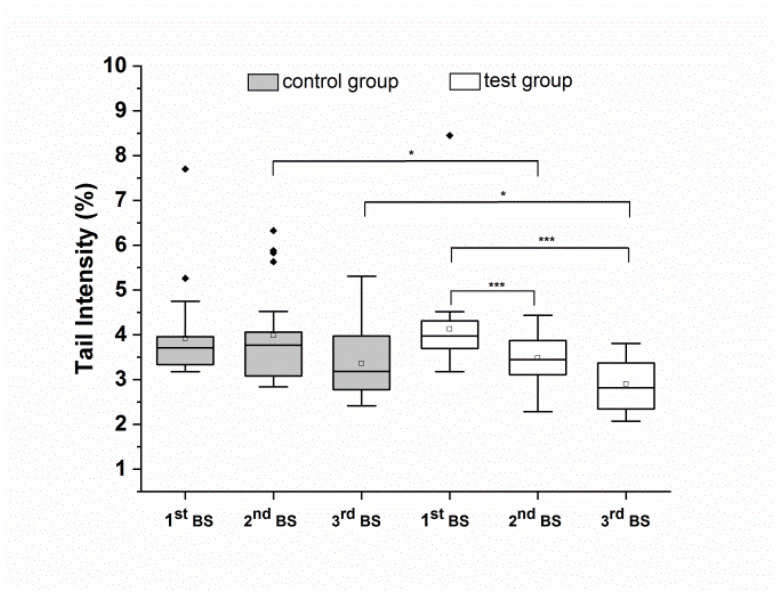
H_2_O_2_-induced DNA strand breaks in peripheral blood lymphocytes isolated from male volunteers (control subgroup, *n* = 23; test subgroup, *n* = 23) after run-in (1^st^ BS), and after four weeks (2^nd^ BS) and eight weeks (3^rd^ BS) of the intervention. Data are expressed as mean tail intensity [%] and SD. Squares represent mean values; horizontal lines within the boxes represent median values; rhombuses represent outliers. BS: blood sampling. * *p* < 0.05, *** *p* < 0.001.

Isolated peripheral lymphocytes from a subgroup of male volunteers were analyzed after H_2_O_2_ challenge (test group: *n* = 23; control group: *n* = 23). Results are presented in Figure 5. After four weeks and eight weeks of food supplement consumption, compared to baseline, H_2_O_2_-induced DNA strand breaks decreased significantly (*p* < 0.001). In contrast, no modulation of DNA damage was detected in the control group throughout the entire study period. Compared to the control group, food supplement consumption led to a significantly greater decrease in H_2_O_2_-induced DNA strand breaks after four weeks (*p* < 0.05) as well as after eight weeks (*p* < 0.05). The TI% of untreated lymphocytes ranged between 0.52–0.6 TI% (unpublished data) and were therefore comparable to TI% of background DNA strand brakes in whole blood.

Table 5 represents the summarized results as numeric values.

## 4. Discussion

Despite broad consumption worldwide, the usefulness of consuming food supplements is frequently criticized. Intake of highly-concentrated antioxidants, like supplements that include vitamins C and E, or carotenoids, even proved to exhibit detrimental health impacts [26,27,28]. Nevertheless, general recommendations for a healthy diet include sufficient consumption of antioxidant-rich fruits and vegetables, and these recommendations are frequently not upheld by the general population. A sensible approach might comprise physiologic supplements containing naturally-composed polyphenolic mixtures, like (concentrated) fruit juices, and micronutrients essential for the proper functioning of the body’s own antioxidative enzyme system. Moreover, a sufficient supply of essential micronutrients and secondary metabolites from plants might support immune cells in advance of extrinsic/intrinsic triggers that require a powerful response. Numerous intervention studies have reported that consuming polyphenol-rich products protects macromolecules against oxidative damage in healthy humans [7,8,29]. However, many of these studies investigated the intake of rather large volumes of polyphenol-rich juices, a practice that does not reflect a normal, suitable daily routine in human nutrition.

We recently conducted an uncontrolled, pilot intervention study with a food supplement based on concentrated aronia juice, which is rich in polyphenols, to test whether this supplement would produce health benefits with consumption of smaller daily quantities. We observed trends towards a decrease in DNA strand breaks and an increase in gene expression of the Nrf2 transcription factor, but these modulations were not statistically significant due to the small sample (*n* = 10 volunteers) [20]. This pilot study (*n* = 10) was designed as single-arm sequential trial, in which the volunteers are presumed to act as their own controls. Confounding factors were not adequately controlled, however. Therefore, we performed a randomized, controlled clinical trial characterized by a larger sample size (*n* = 91 volunteers), an eight-week intervention period, and evaluation of H_2_O_2_-induced DNA strand breaks alongside testing for background and total DNA strand breaks. The aim of the present study was thus to confirm the protective effect, as detected in our pilot study.

Overall, consumption of the aronia-juice-based food supplement (corresponding to a daily intake of 80 mg of total anthocyanins) was associated with significant reductions in background DNA strand breaks compared to baseline. This observation is similar to findings previously observed after eight weeks daily consumption of 750 mL of anthocyanin-rich red fruit juice (corresponding to 274.5 mg/L total anthocyanins); four weeks daily consumption of 700 mL of fruit juice (containing 197.9 mg/L total anthocyanins); and ingestion of a 700 mL “bolus” of red fruit juice (containing 332.0 mg/L total anthocyanins) within 8 h [7,8,29]. In comparison to the background DNA strand breaks, the total DNA damage in the present study was not significantly changed. In the recently conducted pilot intervention study with the aronia juice based food supplement, the decrease in background DNA strand breaks was also more pronounced than in total DNA strand breaks [20]. Previous intervention studies of background DNA strand breaks using comet assay indicated significantly decreased damage after short- and long-term polyphenol-rich coffee consumption [13,14,30]. Since background DNA strand breaks may result from, e.g., endogenous DNA damaging agents and/or incomplete DNA repair, a decrease in DNA strand breaks can be interpreted as increased cell protection.

Fortunately, baseline DNA damage is low under healthy conditions, but this makes detection of any potentially protective effects of an intervention difficult. Our results from an ex vivo H_2_O_2_-challenge of isolated peripheral lymphocytes may be relevant for detecting the potential protective effects of the intervention of interest since the lymphocyte challenge induces an acute oxidative condition that mimics what may occur in a diseased state. Consumption of the food supplement was associated with significant reductions (up to 25%) of H_2_O_2_-induced DNA damage compared to the control. Again, similar findings were previously observed in healthy volunteers after 6 weeks consumption of a wild blueberry drink [12], 4 weeks daily ingestion of 1 L of a mixture of blueberry and apple juice [31], and intake of a single portion of blueberries (300 g) [32]; each of these treatment interventions improved protection against H_2_O_2_-induced DNA damage in isolated lymphocytes. Therefore, regular consumption of antioxidants might exhibit preventive effects that become measurable only under oxidative stress conditions. A recent publication on ROS accumulation in the gut of Drosophila flies, induced by sleep deprivation and ultimately leading to death, impressively demonstrated that antioxidants can counteract oxidative stress in vivo. Moreover, in addition to oxidative stress reduction, oral antioxidants also prevented death and allowed a normal lifespan despite little to no sleep in these flies. Accumulation of oxidative stress in the gut was similarly observed in mice, suggesting that the treatment intervention may have equivalent effects in mammals [33].

Aronia berries are rich in polyphenolic compounds that trigger marked radical-scavenging activity and elevate antioxidant defense via the Nrf2/ARE signaling pathway. Induction of transcript levels of the redox sensor Nrf2 has indeed been previously observed with the food supplement under investigation [20], and others have reported on the increased activities of Nrf2-dependent enzymes (SOD and CAT) after consumption of anthocyanin-rich juices and smoothies [6]. Additionally, micronutrients such as zinc (a co-factor of SOD) and selenium (a co-factor of GPx) are required for their respective enzymes to function and, thus, presumably contribute to the DNA-protective effects observed. Such synergistic mechanism may be supported by the overall low amounts of total anthocyanins, as compared to previous studies applying larger volumes of polyphenol rich juices. A multiple-arm randomized trial would be needed to clearly identify the actions of single ingredients of the supplement. Nonetheless, our results demonstrate that daily consumption of physiological doses of antioxidant food supplements may exhibit beneficial health effects and thus warrant further long-term investigations.

Our study has some limitations. First, although we established a control group, randomized participant assignment to either the intervention or the control group was not blinded. Designing a placebo with color that tastes sweet-and-sour and astringent, and that causes no physiologic effect, is a true challenge. Since our primary endpoints were based on objective measurements of blood characteristics, we consider our approach a suitable compromise. Still, we cannot fully exclude a placebo effect due to a likely difference in the volunteers’ daily expectations of consuming the supplement compared to consuming pure water. Second, H_2_O_2_-induced DNA strand breaks were determined in isolated peripheral blood lymphocytes in the subgroups of male volunteers only due to the laborious experimental setup. However, since no gender-specific effects were observed in all other characteristics tested, we assume that the effects clearly observed for male subjects may be expected in females as well. In previous studies, no differences between men and women were found in any of the DNA damages studied (background DNA strand breaks, FPG-, and endonuclease III-sensitive sites) [34,35].

Taken together, the consumption of the aronia-juice-based food supplement contributes to the maintenance of DNA integrity in healthy volunteers.

## Figures and Tables

**Table 1:** Composition of aronia+ drinking ampule.

	Amount per 25 mL Drinking Ampule	Amount per 2 × 25 mL Drinking Ampule (Daily Study Dosage)
**Vitamin D3**	10 µg	20 µg
**Vitamin B1**	1.1 mg	2.2 mg
**Vitamin B2**	1.4 mg	2.8 mg
**Niacin**	16 mg	32 mg
**Pantothenic acid**	6 mg	12 mg
**Vitamin B6**	1.4 mg	2.8 mg
**Zinc**	4.5 mg	9.0 mg
**Selenium**	50 µg	100 µg
**Aronia juice concentrate**	6.75 g	13.50 g

**Table 2:** Baseline characterization of the volunteers.

	Test Group	Control Group	Test vs. Control *p*-Value
**Number (*n*)**	50	49	**-**
**Sex (women/men)**	25/25	25/24	**-**
**Age (years)**	26.3 ± 6.6	25.1 ± 5.9	0.35
**Weight (kg)**	72.9 ± 13.7	71.5 ± 11.2	0.41
**Height (m)**	1.7 ± 0.1	1.7 ± 0.1	0.75
**BMI (kg/m^2^)**	23.8 ± 3.4	23.6 ± 2.8	0.51

**Table 3:** Background DNA strand breaks (without FPG treatment) in whole blood. Data are expressed as mean tail intensity [%] and SD; BS: blood sampling.

Group	1^st^ BS	2^nd^ BS	3^rd^ BS	2^nd^ vs. 1^st^ *p*-Value	3^rd^ vs. 1^st^ *p*-Value	2^nd^ BS *p*-Value	3^rd^ BS *p*-Value
Control	0.44 ± 0.11	0.40 ± 0.1	0.41 ± 0.1	0.03	0.32		
Test	0.46 ± 0.09	0.39 ± 0.08	0.42 ± 0.1	0.001	0.02		
Test vs. Control						0.46	0.49

**Table 4:** Total DNA strand breaks (with FPG treatment) in whole blood. Data are expressed as mean tail intensity [%] and SD; BS: blood sampling.

Group	1^st^ BS	2^nd^ BS	3^rd^ BS	2^nd^ vs. 1^st^ *p*-Value	3^rd^ vs. 1^st^ *p*-Value	2^nd^ BS *p*-Value	3^rd^ BS *p*-Value
Control	1.24 ± 0.35	1.31 ± 0.35	1.32 ± 0.4	0.32	0.27		
Test	1.27 ± 0.35	1.25 ± 0.34	1.28 ± 0.32	0.75	0.95		
test vs. control						0.39	0.48

**Table 5:** H_2_O_2_-induced DNA strand breaks in peripheral blood lymphocytes isolated from male volunteers (control subgroup, *n* = 23; test subgroup, *n* = 23). Data are expressed as mean tail intensity [%] and SD; BS: blood sampling.

Group	1^st^ BS	2^nd^ BS	3^rd^ BS	2^nd^ vs. 1^st^ *p*-Value	3^rd^ vs. 1^st^ *p*-Value	2^nd^ BS *p*-Value	3^rd^ BS *p*-Value
Control	3.91 ± 0.96	3.98 ± 1.0	3.36 ± 0.74	0.8	0.06		
Test	4.13 ± 0.35	3.48 ± 0.51	2.9 ± 0.57	0.001	0.001		
Test vs. Control						0.05	0.02

## Data Availability

Data of the study are not publicly archived.
